# *In silico* investigation of Alsin RLD conformational dynamics and phosphoinositides binding mechanism

**DOI:** 10.1371/journal.pone.0270955

**Published:** 2022-07-18

**Authors:** Marco Cannariato, Marcello Miceli, Marco Agostino Deriu

**Affiliations:** Polito^BIO^Med Lab, Department of Mechanical and Aerospace Engineering, Politecnico di Torino, Turin, Italy; University of Helsinki, FINLAND

## Abstract

Alsin is a protein known for its major role in neuronal homeostasis and whose mutation is associated with early-onset neurodegenerative diseases. It has been shown that its relocalization from the cytoplasm to the cell membrane is crucial to induce early endosomes maturation. In particular, evidences suggest that the N-terminal regulator of chromosome condensation 1 like domain (RLD) is necessary for membrane association thanks to its affinity to phosphoinositides, membrane lipids involved in the regulation of several signaling processes. Interestingly, this domain showed affinity towards phosphatidylinositol 3-phosphate [PI(3)P], which is highly expressed in endosomes membrane. However, Alsin structure has not been experimentally resolved yet and molecular mechanisms associated with its biological functions are mostly unknown. In this work, Alsin RLD has been investigated through computational molecular modeling techniques to analyze its conformational dynamics and obtain a representative 3D model of this domain. Moreover, a putative phosphoinositide binding site has been proposed and PI(3)P interaction mechanism studied. Results highlight the substantial conformational stability of Alsin RLD secondary structure and suggest the role of one highly flexible region in the phosphoinositides selectivity of this domain.

## 1 Introduction

Alsin is a protein with crucial functions for the homeostasis within neurons. It has been shown that its mutations are associated with the development of motor neuron diseases such as Infantile-onset Ascending Hereditary Spastic Paralysis (IAHSP) [1–3]. In particular, mutations account for the impairment of specific subcellular processes, among which there is Alsin relocalization from the cytoplasm to membrane ruffles [1, 4]. This event is necessary for the subsequent Alsin-dependent activation of Rab5, a guanosine triphosphatase (GTPase), leading to early endosomes maturation [1, 4]. The relocalization process has been demonstrated to be triggered by the activation of Rac1, another GTPase, and has been suggested to be correlated to Alsin interaction with phosphoinositides (PIPs) [5, 6]. These membrane phospholipids are involved in the recruitment of specific proteins to the membrane, thus regulating several cellular events. Among such processes, vesicles formation after membrane ruffling is known to be controlled by the sequential breakdown of phosphatidylinositol 3,4,5-triphosphate [PI(3,4,5)P_3_] to phosphatidylinositol 3-phosphate [PI(3)P] [7, 8]. While PI(3,4,5)P_3_ has been observed mainly before vesicle formation, together with Rac1 recruiting on the membrane, maturing vesicles are characterized by the colocalization of PI(3)P and Rab5 [9]. Interestingly, Alsin is one of the proteins suggested to regulate the transition from Rac1-PI(3,4,5)P_3_ to Rab5-PI(3)P [9]. In particular, Alsin N-terminal domain, i.e. the regulator of chromosome condensation 1 (RCC1)-like domain (RLD), is essential for membrane localization of the whole protein and is suggested to have a major role in the association with matured vesicles after Rac1 detachment [6]. Moreover, a recent study highlighted that Alsin RLD and pathogenic missense mutants bound specific PIPs with different affinities. In particular, a greater affinity towards PI(3)P than PI(3,4,5)P_3_ was suggested, whereas no significant difference in the patterns of lipid affinity was observed between WT and mutated RLD [1]. In this context, the investigation of molecular mechanisms driving the different affinities of RLD towards PIPs is important to understand Alsin membrane association and how it might be impaired in pathological conditions. Computational molecular modeling techniques, such as molecular dynamics (MD), have been used previously to study Alsin relocalization-related events, i.e. the interaction with Rac1, and PIPs binding to other proteins [10–12]. However, the first step to investigate the molecular mechanisms associated with Alsin RLD interaction with PIPs is to obtain a representative 3D structure of this domain. Alsin RLD has been already predicted to fold into a seven-bladed β-propeller both through homology modeling and AlphaFold (AF) predictor. These predictions highlighted that the propeller is interrupted by an intrinsically disordered region (IDR), which was demonstrated to be necessary for a proper oligomerization of the protein [1, 13–15]. However, the molecular basis of Alsin RLD affinity and selectivity towards PIPs remains unclear, therefore this work aims at studying Alsin RLD exploiting computational molecular modeling tools. In greater detail, Alsin conformational dynamics was investigated through MD techniques and a putative lipid-binding site was identified to study the interaction with PI(3)P and PI(3,4,5)P_3_. The results will provide an overview of Alsin RLD dynamics and insights into a potential PI(3)P association mechanism. Furthermore, the putative molecular basis of Alsin RLD selectivity towards PIPs will be reported.

## 2 Materials and methods

### 2.1 Molecular dynamics of Alsin RLD

Since Alsin RLD structure has not been experimentally resolved yet, three different 3D models were compared using Molecular Operating Environment (MOE) software [16]. Two of them were developed according to protocols previously described by Soares et al. [13] and Sato et al. [1], therefore in the following will be called RLD^SO^ and RLD^SA^, respectively. The third one was retrieved from AF Protein Structure Database, neglecting the IDR (https://alphafold.ebi.ac.uk/entry/Q96Q42, aa. 1–225 and 520–690), in the following called RLD^AF^. The above-mentioned three models, i.e., RLD^SO^, RLD^SA^, and RLD^AF^ were compared considering their secondary structure, RMSD, and Z-score. The RMSD was computed between the alpha carbons of the models after their superimposition. The Z-score of a model is the energy deviation of its structure from a set of random configurations, therefore can be considered as a measure of the overall quality of a model [17, 18]. The Z-scores of the investigated 3D models were obtained using ProSA webserver [19]. Among the abovementioned models, RLD^AF^ was considered for the following analysis due to its lower Z-score.

Atomic positions for Alsin RLD were retrieved from AF Protein Structure Database as described before. Since the IDR was not considered, the structure was composed of two chains, i.e. chain A (aa. 1–225) and B (aa. 520–690), interacting through non-covalent interactions. The protonation state was adjusted according to a pH of 7.4 using PROPKA [20, 21], while AMBER ff99SB-ILDN force field was employed to build the topology [22]. A dodecahedron box was defined imposing a minimum distance of 1 nm between box edge and protein. Explicit TIP3P water was used to solvate the box [23], then Na^+^ and Cl^-^ ions were added to neutralize the total charge and reach a physiological concentration of 0.15 M. The energy of the system was minimized through 2000 steps of steepest descend algorithm, then three replicas were obtained with the subsequent procedure. A first 200 ps simulation in NVT ensemble was followed by a 500 ps equilibration in NPT ensemble, both under position restraint of alpha carbons. Temperature coupling was performed using the modified Berendsen thermostat [24] at a reference temperature of 300 K (τ = 1 ps), while pressure coupling was carried out through Berendsen barostat at 1.0 bar (τ = 5 ps). Then, a 500 ns MD simulation, without any position restrain, was produced in NPT ensemble integrating the equation of motion every 2 fs using leapfrog algorithm. Particle mesh Ewald method was employed to treat electrostatic interactions, together with potential switching starting at 1.0 nm and short-range cutoff at 1.2 nm. Van der Waals interactions were treated with potential switching starting at 1.0 nm and short-range cutoff at 1.2 nm. All simulations were carried out in GROMACS 2020.4 [25].

### 2.2 Molecular dynamics of RLD-PIP complexes

To generate RLD-PIP complexes, initial coordinates of Alsin RLD were obtained from the equilibrium trajectories of RLD alone. Possible binding sites were identified through MOE site finder tool and were evaluated by Propensity for Ligand Binding (PLB) metric, which can be used to rank sites considering their geometry, accessibility, and amino acid composition [26]. Surface properties of the domain were investigated through MOE software by calculating protein patches, i.e. clusters of neighboring residues sharing similar electrostatic and hydrophobic properties. Among the possible binding pockets, the one with the second-highest PLB was considered since it colocalized with a positive surface patch. The structures of PI(3)P and PI(3,4,5)P_3_ heads were built in MOE, keeping two carbons of glycerol backbone as done previously (S1 Fig in S1 File) [27]. To obtain RLD-PI(3)P and RLD-PI(3,4,5)P_3_ complexes, the protonation state of both ligands was adjusted in MOE according to a pH of 7.4, then the PIPs heads were docked on the abovementioned site. Receptor atoms were kept fixed, 1000 poses were generated and scored with London dG scoring function, then the best 30 poses were refined and evaluated through GBVI/WSA dG scoring function. Finally, the best pose of each ligand was considered to obtain the two complexes. The docking procedure was carried out using MOE software.

The stability of the obtained RLD-PI(3)P and RLD-PI(3,4,5)P_3_ complexes was investigated through MD simulations. To this purpose, PIPs topologies were built using the General Amber Force Field (GAFF) [28] and the AM1-BCC charge method [29] as done previously [30, 31]. For each complex, the setup described in section 2.1 was employed to prepare, energy minimize, and equilibrate the system before performing three 200 ns long MD replicas.

### 2.3 Simulation analysis

The structural stability of each system (free RLD, RLD-PI(3)P, and RLD-PI(3,4,5)P_3_) during the MD simulations was evaluated through the root-mean-square deviation (RMSD) throughout the trajectory of alpha carbons from their initial coordinates. Regarding RLD alone, from the visual inspection of RMSD plots (S2 Fig in S1 File) the last 400 ns of trajectories were considered in the following analysis, while the whole RLD-PIPs simulations were considered since the starting configuration of RLD was derived from equilibrium trajectories. The secondary structure probability at the end of free RLD simulations was evaluated through STRIDE software package [32]. In particular, the last 50 ns of each replica were concatenated and snapshots were extracted every 100 ps. Then, the secondary structure of all configurations was obtained and then the results averaged [33]. The root-mean-square fluctuation (RMSF) of Cα was computed to identify the most mobile regions of RLD within each replica, both alone and in presence of PIPs, then the results averaged.

The stability of PIPs binding poses was evaluated considering the RMSD from the initial position, least-square fitting the trajectory on protein Cα. Then, the binding energy was computed through the Molecular Mechanics Generalized Born Surface Area (MMGBSA) method [34] using parameters as suggested by previous literature [35, 36]. In particular, five equally spaced 100 ps windows were considered in the last 50 ns of each MD trajectory. Since PI(3,4,5)P_3_ detached during one replica, the last 50 ns before the displacement from the binding site were considered in that simulation. Due to its observed dynamics, an interesting region was loop L3 (aa. 641–655), therefore its conformational dynamics, in presence and absence of PIPs, has been described using two coordinates. The first is the RMSD of L3 Cα from their position at the end of free RLD trajectories. The second is the distance between the putative binding pocket, represented by the center of mass of residues 619, 640, and 677, and L3, represented by the center of mass of residues 645 to 647. The choice of the specific residues derived from the analysis of binding energy decomposition (section 3.3).

GROMACS built-in functions were employed to compute RMSD and RMSF. Python libraries and custom-made scripts were used to analyze the conformational dynamics and prepare data plots [37]. Visual molecular dynamics (VMD) [38] and MOE were used to render 3D representations of the proteins.

### 2.4 Protein motif similarity analysis

To investigate the ability of the selected pocket to bind a phosphate ligand, similar 3D arrangements of residues (i.e. motifs) were searched within the Protein Data Bank (PDB) using ASSAM web server [39]. Indeed, similar motifs may be correlated with similar functions [40]. For this purpose, the following procedure was employed. First, the last 50 ns of the RLD-PIP replicas were concatenated and clustered with gromos method and a cutoff of 0.1 nm. Least-square fitting was performed on residues within 1 nm from the ligand in the equilibrium structure of RLD-PIP complex. Then, the centroids of clusters containing more than 5% of the total frames were extracted. The motifs provided as input to ASSAM were generated from such centroids considering only the residues interacting with the ligand, which were detected using PLIP library [41]. To discard buried motifs, ASSAM results (also recalled as hits in the following) were then filtered according to the solvent accessible surface area (SASA) of the identified sites. A threshold equal to the 20% of the average SASA pertaining to query motifs was considered to filter ASSAM results. Hence, proteins characterized by motifs similar to the target but barely exposed or not exposed to the solvent are not likely able to interact with a ligand dispersed in the solvent and then they should not be considered as a hit. For those hits above the threshold, the ability to interact with the PIP was evaluated by docking the phosphoinositide to all of them. Then, ASSAM hits were ranked according to the docking score, where a lower score indicates a greater affinity for the PIP. Docking was performed with AutoDock Vina, setting the exhaustiveness level to 64 and using the sites identified by ASSAM to define the binding pocket with AutoGridFR (padding = 2Å) [42, 43].

## 3 Results

### 3.1 Alsin RLD model selection

Existing Alsin RLD 3D models, namely RLD^SO^, RLD^SA^, and RLD^AF^, were compared to select the best initial structure to investigate Alsin RLD conformational dynamics. It was possible to observe an overall consensus in terms of secondary structure between the three models, except for some differences discussed in the following. Within the first 40 residues, RLD^SA^ had a different secondary structure compared to the other models, that were characterized by coiled-coil N-terminals. Moreover, high RMSD values were obtained within the region spanning residues 120–150. Here, RLD^SO^ presented a β-wedge protruding from the third blade, which is known to be crucial for RCC1 GEF activity [44]. However, Alsin RLD showed no detectable GEF functions [6, 45]. In addition, improper folding of the outer *β*-strand within the third blade was observed in RLD^SA^ (S3 Fig in S1 File). In S4 Fig in S1 File a detailed representation of 3D models secondary structure and per-residue RMSD is shown. Furthermore, RLD^AF^ Z-score was the closest to the distribution of values across experimentally resolved protein from PDB (S5 Fig in S1 File). Therefore, RLD^AF^ has been selected for further analysis.

### 3.2 RLD^AF^ model conformational dynamics

**Fig 1:**
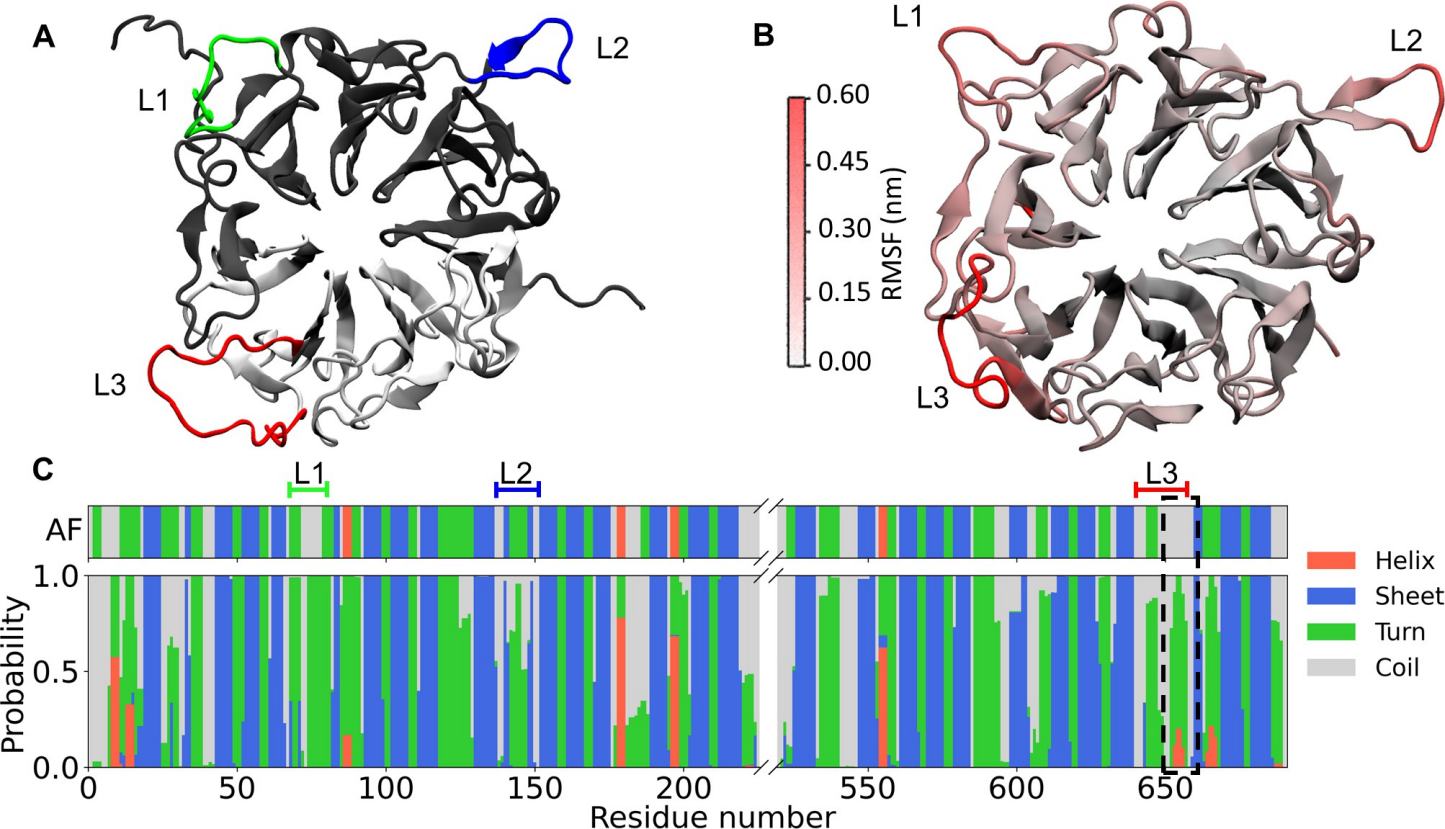
Alsin RLD structural refinement. (**A**) RLD^AF^ model is characterized by three loops protruding from the propeller, i.e. L1 (green), L2 (blue), and L3 (red). Chain A and B are colored in grey and white, respectively. (**B**) Refined Alsin RLD model. Residues are colored according to their RMSF to highlight in red the most flexible regions. For visualization purposes, the highly flexible N-terminal, aa. 220–225, and aa. 520–525 are not shown. (**C**) Secondary structure probability for each residue compared to the initial structure of RLD^AF^. The region with increased turn probability after the dynamics is highlighted.

The RLD^AF^ structure, characterized by a seven-bladed β-propeller region discontinued by loops L1-3 (Fig 1A), was stable throughout the overall MD simulation, with a maximum RMSD lower than 1 nm (S2 Fig in S1 File). To identify the most flexible RLD regions, Cα RMSF analysis was carried out. Low values were obtained within the propeller, while the most flexible regions were the disordered ones (N-terminal, L1-L3, aa. 220–225, and aa. 520–525), as is shown in Fig 1B. The overall secondary structure of Alsin RLD was conserved during MD simulations, however, a region within L3 showed a tendency toward turn arrangement, and with a lower probability, toward a helix (Fig 1C). This propensity involved only a part of L3, therefore it did not lower the overall flexibility of L3. The substantial stability of the propeller arrangement was also confirmed by a cluster analysis of the last 50 ns of replicas, using a Cα RMSD cutoff of 0.15 nm and gromos method, which highlighted the presence of only one cluster. The cluster centroid was considered as the refined RLD model.

### 3.3 Identification of PIP binding site

**Fig 2:**
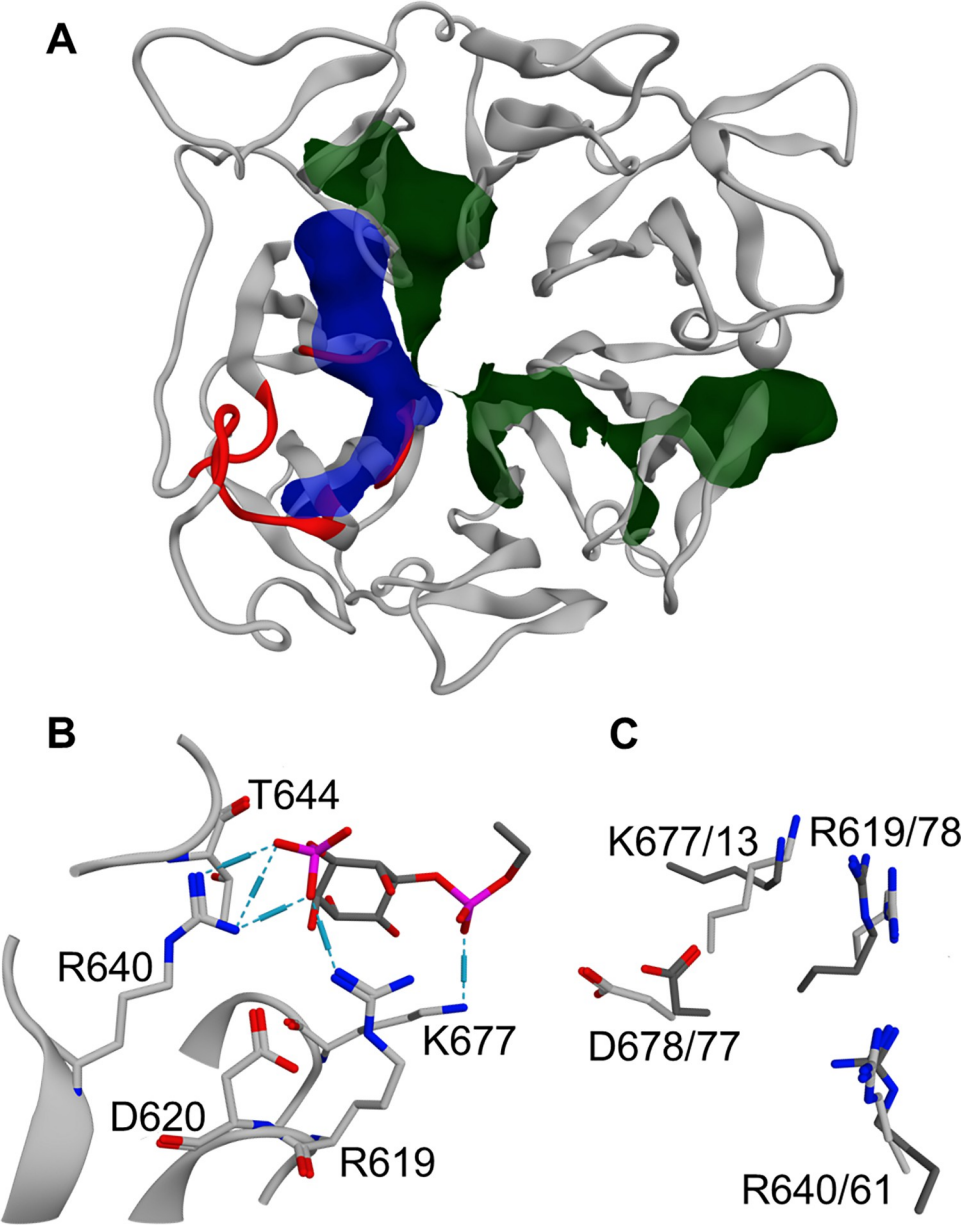
Identification of a putative PIP-binding site in Alsin RLD. (**A**) Visual rendering of the investigated binding pocket. The positive and hydrophobic surface patches are shown in blue and green, respectively, while residues forming the binding site are colored in red. (**B**) Binding pose of PI(3)P at the end of the dynamics, where interactions between two atoms are represented by dashed lines. The strength of an interaction is visually represented by the length of the cylinder. (**C**) Superimposition of RLD query residues (light grey) and the corresponding amino acids of the best hit found through ASSAM (dark grey). In the figure, residue numbers are reported according to the scheme *N*_*query*_/*N*_*hit*_.

A known methodology to identify putative PIP-binding sites is the electrostatic characterization of the protein surface [11]. Indeed, PIPs are negatively charged phospholipids and their binding pockets in other proteins are characterized by a cluster of positively charged residues [11, 27, 46]. Therefore, to define a possible PIP-binding site for Alsin RLD, the surface properties of the refined model were investigated by calculating protein patches. Hydrophobic patches were distributed over all RLD surface and were characterized by the highest areas (S1 Table in S1 File). Interestingly, one positively charged cluster of residues could be found near L3, between two hydrophobic areas. This result was confirmed by computing the surface potential through the APBS software [47], which highlights a negatively charged area in correspondence to L3 (S6 Fig in S1 File). Possible binding sites were then identified using MOE site finder tool and evaluated by their PLB (S2 Table in S1 File). While the highest PLB corresponded to the central cavity of the propeller, the second-highest PLB site partially colocalized with the abovementioned positive patch and L3, as is shown in Fig 2A. Therefore, this pocket has been further analyzed in terms of its PIPs-binding properties.

Since experimental evidence on Alsin RLD suggested a greater affinity towards PI(3)P than PI(3,4,5)P_3_ [1], the selected site has been investigated in terms of the ability to interact with these phosphoinositides. RLD-PIPs complexes were characterized by ligand RMSD, obtained fitting the system (protein + ligand) on protein Cα, lower than 1 nm during almost all the MD simulations, with the only exception of one replica in RLD-PI(3,4,5)P_3_ complex, where the ligand detached from the binding site (S7 Fig in S1 File). A cluster analysis on protein Cα and PIP atoms over the last 50 ns of dynamics (cutoff = 0.15 nm, method = gromos) highlighted the presence of only one cluster in RLD-PI(3)P system. On the other hand, different clusters were obtained in the case of PI(3,4,5)P_3_. Therefore, between the two ligands, PI(3)P showed greater stability of its binding mode, characterized by several interactions between its phosphate groups and Alsin basic amino acids, in particular R619, R640, and K677 (Fig 2B). It is worth mentioning that, in the observed pose, the glycerol backbone arrangement was compatible with the presence of two acyl chains inserted into a membrane.

Given the stability of the RLD-PI(3)P system, PI(3)P binding pose at the end of the dynamics was used to identify similar motifs through ASSAM web server. Among the obtained results, the lowest docking score corresponded to a deoxyadenosine kinase (PDBid 2JAQ [48]). In the crystal structure, the residues identified by ASSAM are located in the active site and the positively charged ones interact with the phosphate groups of a deoxycytidine triphosphate. One acidic residue was used by ASSAM to describe the binding pocket, however it shows no contact interactions with the ligand in both RLD and deoxyadenosine kinase. The superimposition of these amino acids with the corresponding residues of Alsin RLD is shown in Fig 2C. Moreover, in the experimental structure of the protein characterized by the second-lowest docking score (PDBid 3U9S [49]), the identified residues interact with a phosphate group of coenzyme A. Therefore, motifs similar to the amino acids composing the putative RLD PIP-binding pocket are involved in interactions with phosphate groups. Thus, these results strengthen the hypothesis that the selected site in Alsin RLD might be involved in PI(3)P-binding.

**Fig 3:**
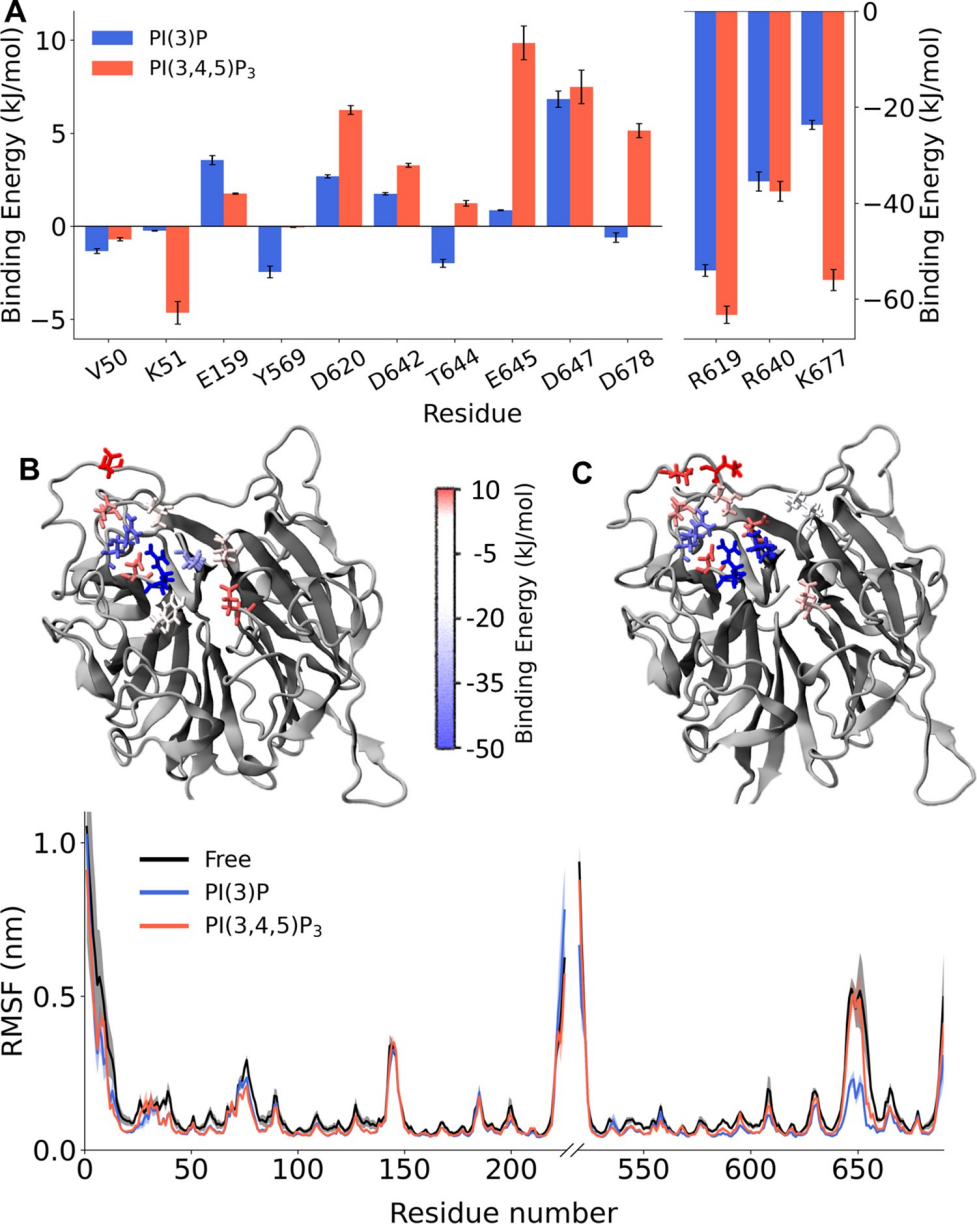
Comparison of RLD-PIPs interaction. (**A**) Decomposition of PI(3)P and PI(3,4,5)P_3_ binding free energy, where error bars represent standard deviations. (**B**) Visual rendering of PI(3)P binding energy decomposition. (**C**) Visual rendering of PI(3,4,5)P_3_ binding energy decomposition. (**D**) Comparison of RMSF values in absence and presence of PIPs. The shaded areas represent the standard deviation of the RMSF between the simulations. Loop L1, L2, and L3 are highlighted within the RMSF profile.

Alsin RLD affinity towards PIPs has been investigated computing their binding free energy. While RLD-PI(3)P interaction was characterized by an energy of -81.6 ± 2.3 kJ/mol, positive energy was obtained in the case of RLD-PI(3,4,5)P_3_ (47.4 ± 7.3 kJ/mol). Despite this remarkable difference, the decomposition of binding free energy showed stronger interactions with three basic amino acids, i.e. R619, R640, and K677, in case of PI(3,4,5)P_3_. At the same time, however, D620, D642, T644, E645, and D678 were repulsing of PI(3,4,5)P_3_, while lower energies were observed in presence of PI(3)P (Fig 3A). These residues were neighboring the abovementioned basic amino acids and, interestingly, the strongest repulsions were observed for E645 and D647, localized on L3 (Fig 3B and 3C). Therefore, the presence of multiple phosphate groups was associated with the repulsion of PI(3,4,5)P_3_ by several residues, leading to an overall destabilization of the complex. At the same time, the RMSF profiles showed that only the presence of PI(3)P resulted in a stabilization of L3, while in the case of PI(3,4,5)P_3_ almost no difference from RLD alone could be observed (Fig 3D).

### 3.4 Conformational dynamics of L3

**Fig 4:**
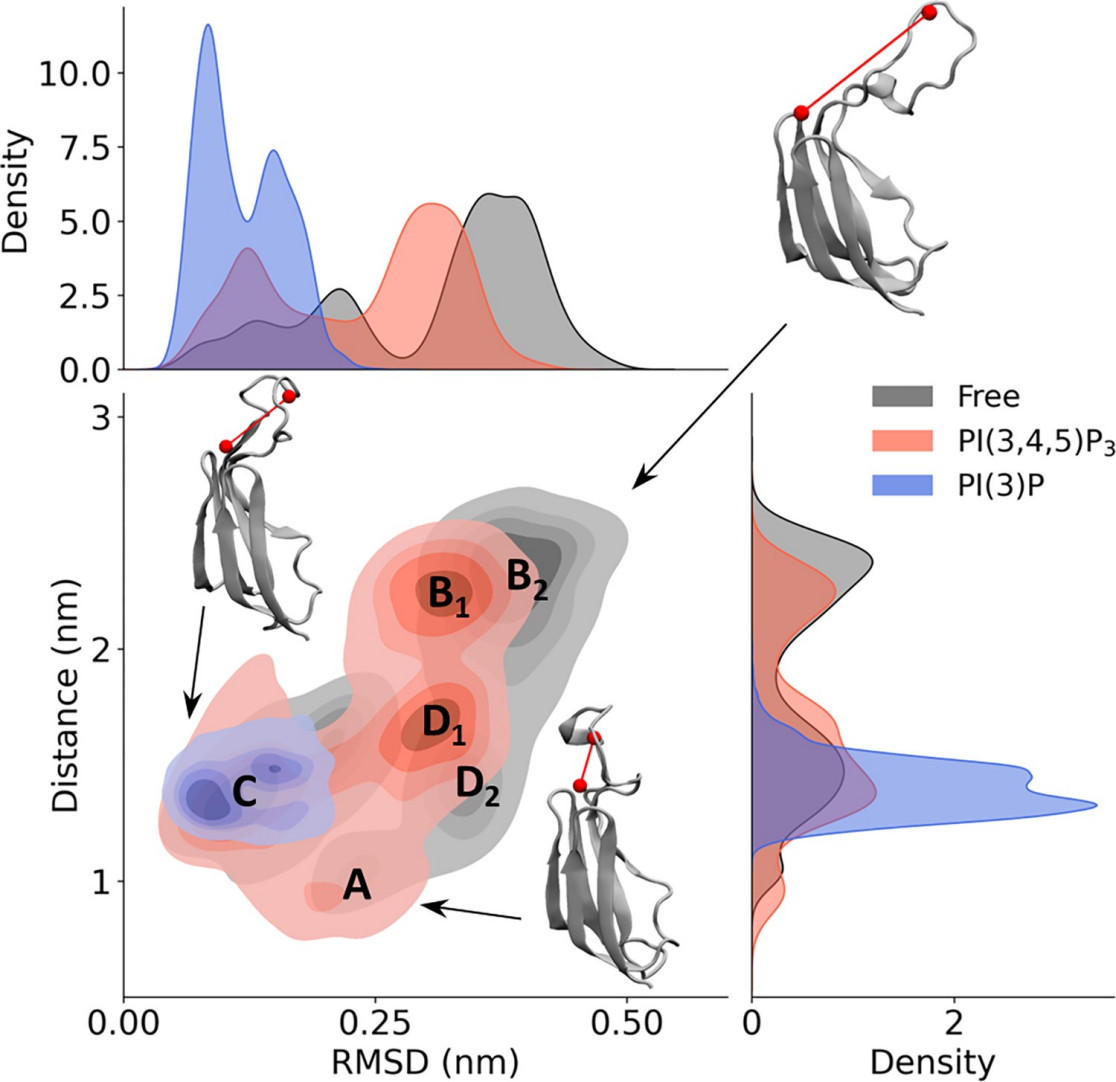
Analysis of L3 conformational dynamics. L3 dynamics has been described as RMSD from its position in the refined RLD model and distance from the investigated binding site. The probability distribution in the case of RLD alone, PI(3)P-bound RLD, and PI(3,4,5)P_3_-bound RLD is shown in black, blue, and red, respectively. Representative configurations of L3 are shown highlighting its distance from the binding pocket in red.

To characterize the fluctuations of L3 and how those fluctuations are influenced by PIPs, L3 conformational dynamics has been described in terms of RMSD and its distance from the putative binding pocket. Fig 4 shows the probability density of L3 conformational states on a RMSD-distance plane. Darker areas correspond to higher values of the probability density functions. Free, PI(3)P-bound, and PI(3,4,5)P_3_-bound systems are highlighted in different colors and the states of interest are referred to with letters from A to D. Moreover, the marginal probability distributions over L3 RMSD and the distance are shown. The L3 RMSD has been measured referred to its conformation in the refined RLD model and provides information about L3 conformational rearrangements. Higher RMSD values correspond to higher deviation from the reference configuration. The distance has been computed between the positively charged amino acids (R619, R640, K677) within the PIP putative binding pocket and the L3 acidic residues (E645 and D647) repulsing PI(3,4,5)P_3_ (Fig 3A). Higher distances are interpreted as increased accessibility of the putative binding site, whereas lower distances as decreased accessibility due to repulsive interactions strongly reducing the binding affinity.

Free and PI(3,4,5)P_3_-bound RLD were characterized by similar L3 conformational dynamics, which explored configurations with different distances from the identified binding site. In particular, in state A the L3 acidic residues are closer to the putative binding site (distance ~ 1nm), therefore the accessibility of the binding pocket is reduced by repulsing interaction with a possible phosphate ligand. On the other hand, in states B_1_ and B_2_ repulsing interactions are less probable due to distances above 2nm, thus the accessibility of the putative site to negatively charged ligands is higher. Interestingly, state B_1_, explored in PI(3,4,5)P_3_-bound system, is characterized by lower RMSD values compared to B_2_, sampled in the case of free RLD. The same difference is present between states D_1_ (PI(3,4,5)P_3_-bound RLD) and D_2_ (free RLD), which are characterized by distances between 1 nm and 2 nm, with D_1_ exhibiting higher values. Therefore, PI(3,4,5)P_3_ seems to limit L3 conformational rearrangements, as can be appreciated by the RMSD marginal distribution. Finally, the lowest RMSD values were found in state C, which is representative of the L3 configuration in the refined RLD model. Notably, states C and D_1,2_ are characterized by similar distances but different RMSD values, thus highlighting conformational rearrangements that do not alter the L3 distance from the identified site. Based on these results, L3 conformational dynamics might indirectly alter the pocket accessibility, suggesting a role of L3 in the regulation of ligand binding. Interestingly, only state C was sampled in presence of PI(3)P, which stabilized distances between 1.35 nm and 1.5 nm from the putative binding site. At the same time, low variation in RMSD values highlights that PI(3)P-binding reduced L3 conformational rearrangements. Given the higher affinity of this ligand to Alsin RLD and L3 stabilization in presence of PI(3)P, a relationship between L3 configuration and RLD membrane association might be suggested.

## 4 Discussion

A detailed knowledge of Alsin structure, possibly characterized by atomic resolution data, is crucial for a proper understanding of Alsin biological functions. Nevertheless, experiments have shown to be limited in producing such knowledge. In this context, computational molecular modeling has already demonstrated the potential to fill this gap, as in the case of the DH/PH domain, recently investigated through MD techniques [10]. In this work, we have investigated the conformational dynamics of Alsin RLD and the potential binding and interaction mechanisms between RLD and PIPs. Such interaction, particularly with PI(3)P, is thought to play a major role in Alsin membrane association, a process impaired in neurodegenerative diseases such as IAHSP [1–3, 5, 50]. It is worth mentioning that, in this work, the RLD intrinsically disordered region was not modelled as that region was suggested to mediate protein-protein interaction rather than membrane association [15]. Moreover, the sole RLD was investigated since experimental evidence demonstrated that the RLD is not involved in Alsin tetramerization [1] and that the affinity towards PI(3)P persists both in the complete tetrameric form [5] and considering only the RLD [1]. Therefore, the PIP binding site within the RLD is probably accessible in the tetrameric Alsin. Based on the above-mentioned observations, these choices allowed to limit the computational effort, yet with minimal influence on the obtained results.

Results from the analysis of surface electrostatic properties and possible binding pockets highlighted the presence of a high-PLB site colocalized with a positive patch (Fig 2A). This site has some conformational features shared among all three considered homology models. However, in RLD^SA^ and RLD^SO^, the residues corresponding to L3 do not extend outside the propeller as in the refined RLD^AF^ (S8 Fig in S1 File). Moreover, residues A25, G26, and S27 of RLD^SA^ and RLD^SO^ are in a different position than in RLD^AF^, as expected given the higher RMSD values of the first 40 N-terminal residues in three models (section 3.1). Since the location of the PIP-binding region within Alsin RLD is still unknown, this pocket was tested as a putative PIP-binding site by analyzing its ability to interact with PI(3)P and PI(3,4,5)P_3_. Consistently with previous experimental results suggesting a lower affinity for PI(3,4,5)P_3_ [1], RLD-PI(3)P complex was characterized by negative binding energy, while PI(3,4,5)P_3_ was associated with positive energy. Moreover, the equilibrium configuration of RLD-PI(3)P complex (Fig 2B) was employed to search similar 3D arrangements of residues on experimentally resolved protein structures. Indeed, similar 3D arrangements of residues could be related to similar functions, despite the absence of sequence identity [40]. Interestingly, the identified residues of the first two best-scoring hits were interacting with phosphate groups in their crystal structures. These results suggest that the analyzed site might be involved in PI(3)P interaction and, therefore, membrane association of the whole domain.

The identification of the putative PIP-binding pocket within Alsin RLD was followed by the investigation of molecular mechanisms underlying its interaction with PI(3)P and PI(3,4,5)P_3_. In the selected site, the presence of acidic residues neighboring the positive ones originated repulsing interactions, which were stronger in case of PI(3,4,5)P_3_ (Fig 3A–3C). In particular, the strongest repulsive forces were located in L3, whose fluctuation was reduced only in presence of PI(3)P (Fig 3D). These results suggest that Alsin RLD selectivity towards PI(3)P might be L3-mediated. At the same time, the analysis of L3 conformational dynamics has shown that its fluctuations are characterized by different distances from the putative binding site. Therefore, the accessibility of this site might depend on the configuration of this loop. When L3 moves closer to the pocket, the presence of the negatively charged E645 and D647 may reduce the accessibility of this region to phosphoinositides. Interestingly, PI(3)P stabilized a single configuration of L3, while in presence of PI(3,4,5)P_3_ the exploration of its conformational space was similar to free RLD (Fig 4). Since such stabilization was observed only in presence of PI(3)P, which showed greater affinity for Alsin RLD, it could be hypothesized a role of this region in the membrane association. Indeed, loops extending from other propellers are known to play a major role in membrane binding by inserting deeply into the membrane itself [12, 46]. Further analysis should be undertaken to investigate this possible function of L3 within Alsin RLD, such as modeling RLD interaction with a PI(3)P-containing membrane as done in recent literature [51].

## 5 Conclusions

The molecular mechanisms driving Alsin physiological and pathological functions are in great part still unknown. However, insights into the nanoscale phenomena associated with this protein represent the first step towards a proper understanding of Alsin-related pathologies, such as IAHSP. Nonetheless, the structures of Alsin domains have not been experimentally resolved yet. In this framework, this study provides a refined and representative 3D structure for Alsin RLD and identifies a putative PIP-binding site. The results obtained from the computational analysis of RLD affinity for PI(3)P and PI(3,4,5)P_3_ were consistent with previous experimental assays [1] and suggest an L3-mediated selectivity towards PI(3)P. Moreover, the stabilization of L3 conformation might suggest its role in membrane association. To further elucidate the Alsin RLD membrane-binding mechanism, additional *in silico* and *in vitro* studies will be necessary.

## Supporting information

S1 File Figures and tables to support “material and methods” and “results” sections of this paper. (PDF)
