# Supplementary material for: In silico investigation of Alsin RLD conformational dynamics and phosphoinositides binding mechanism
Source: PLoS One. 2022 Jul 18;17(7):e0270955. doi: 10.1371/journal.pone.0270955 (PMC9292110; doi:10.1371/journal.pone.0270955)
Supplement: S1 File — (PDF) [file pone.0270955.s001.pdf]

# *In silico* investigation of Alsin RLD conformational dynamics and phosphoinositides binding mechanism

**Marco Cannariato[1], Marcello Miceli[1] and Marco Agostino Deriu[1*]**

[1] PolitoBIOMed Lab, Department of Mechanical and Aerospace Engineering, Politecnico di Torino, 10129 Turin, Italy

***Correspondence:**
marco.deriu@polito.it

## *Supporting Information*

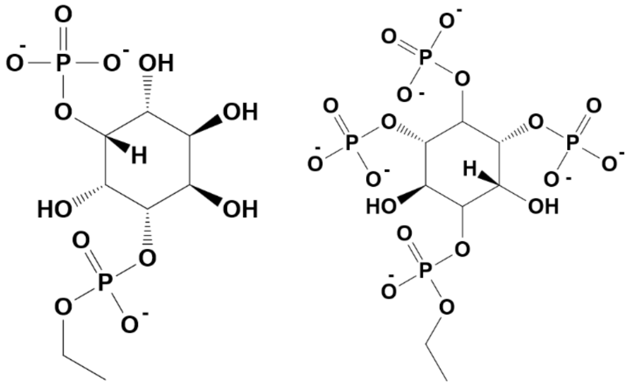


**Fig S1.** Depiction of PI(3)P (left) and PI(3,4,5)$P_3$ (right) molecules. The protonation state has been adjusted in MOE.

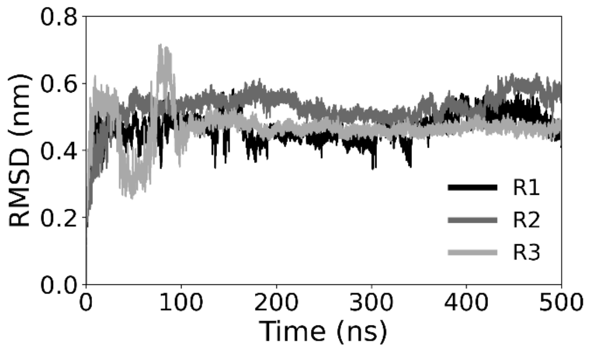


**Fig S2.** RMSD of RLD$^{AF}$ Cα with respect to the initial configurations. The three replicas are highlighted with different colors.

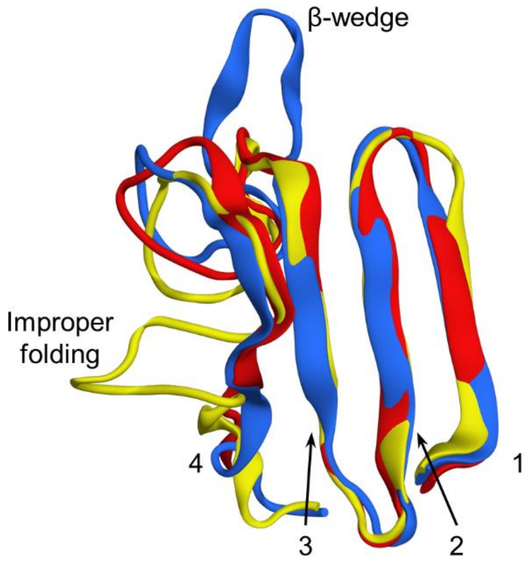


**Fig S3.** Superimposition of the third blade of RLD$^{SO}$ (blue), RLD$^{SA}$ (yellow), and RLD$^{AF}$ (red). The strands are numbered from the center to the outer surface of the propeller. It is possible to observe the improper folding of the outer strand in RLD$^{SA}$ and the $\beta$-wedge extending between the third and fourth strands in RLD$^{SO}$.

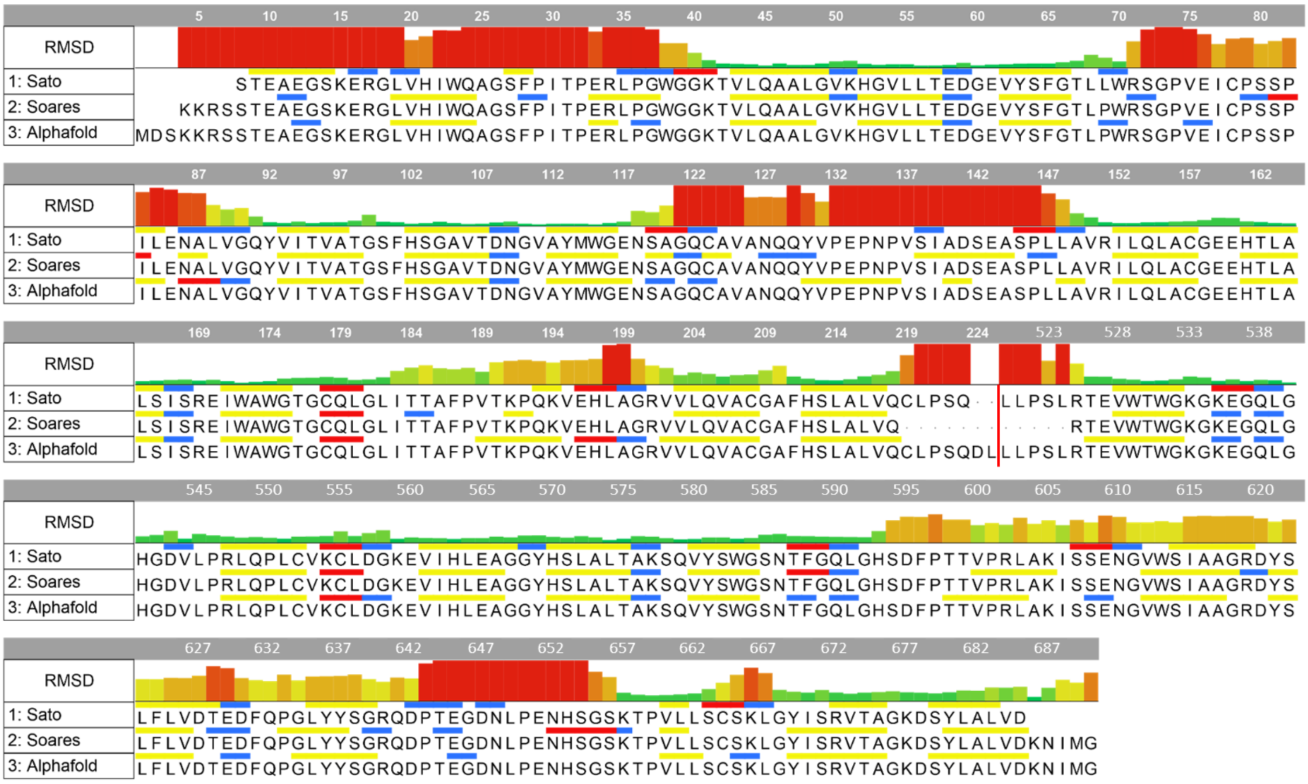


**Fig S4.** Alignment of the three models showing their secondary structure and the RMSD after superimposition. Helices, $\beta$-strands, and turns are highlighted in red, yellow, and blue, respectively. The red line divides chain A (aa. 1-225) from chain B (520-690) in the models. The maximum bar height corresponds to an RMSD $\geq$ 8Å (red), while the minimum bar height corresponds to 0Å (green).

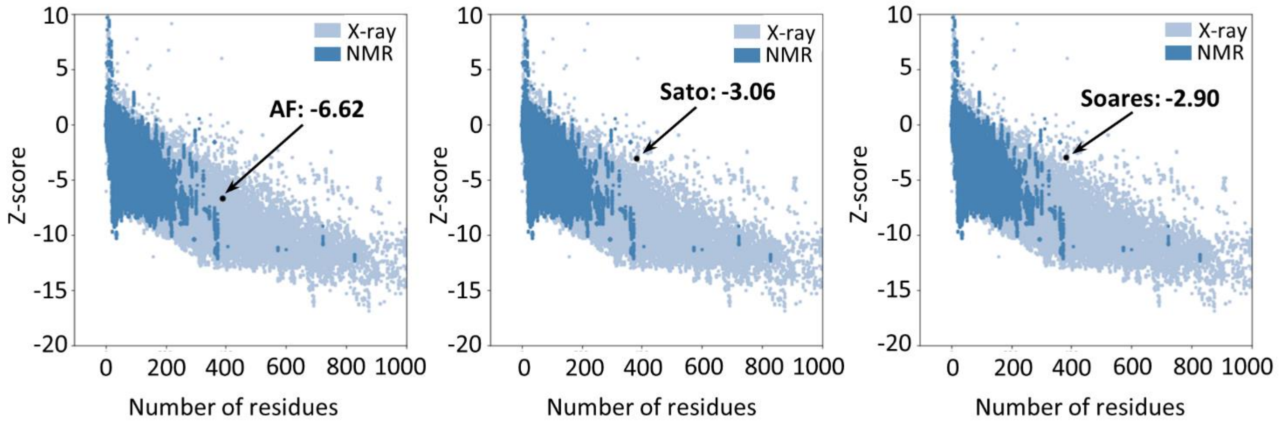


**Fig S5.** Visual representation of Z-score against the number of residues. Experimentally resolved structures from Protein Data Bank are represented as dark (NMR) or light (X-ray) blue dots, while RLD models as black dots.

**Table S1.** Surface patches of the refined RLD model. For each patch, its type (hydrophobic, positive, or negative), area, and the residues composing it are reported. Only patches with an area greater than 100 Å$^2$ are reported. Patches represented in Fig 2 are highlighted in yellow.

| | Type | Area (Å$^2$) | Residues |
|---|---|---|---|
| 1 | hyd | 170 | I562 HIE563 L574 A576 W613 L625 P633 Y669 |
| 2 | hyd | 170 | C178 A210 F211 K533 K535 P546 L548 Y569 R619 |
| 3 | hyd | 160 | Y92 I94 D108 R151 L153 L165 I167 |
| 4 | pos | 120 | V50 K51 F101 F588 R619 R640 K677 D678 |
| 5 | neg | 120 | S142 E143 A144 K194 E196 |
| 6 | hyd | 100 | V50 K51 T67 P69 F101 E117 R619 K677 |
| 7 | hyd | 100 | P36 P74 I77 C78 I83 |
| 8 | neg | 100 | S607 E609 E629 D630 |

**Table S2.** Definition of the sites identified through MOE site finder tool. For each site with positive PLB, residues and PLB are reported. The putative PIP-binding site is highlighted in yellow.

| Site | PLB | Residues |
|---|---|---|
| 1 | 5.19 | Q45 A46 A47 L48 G49 V50 T95 V96 A97 T98 G99 S100 Q154 L155 A156 C157 G158 E159 Q205 V206 A207 C208 G209 A210 F211 H563 L564 E565 A566 G567 G568 S614 I615 A616 A617 G618 R619 R672 V673 T674 A675 G676 K677 |
| 2 | 1.38 | A25 G26 S27 G618 R619 D620 Y621 Y637 R640 Q641 D642 P643 T644 E645 G646 G676 K677 D678 |
| 3 | 1.08 | S64 F65 G66 T67 L68 P69 R71 S72 G73 P74 P79 S81 P82 I83 L84 E85 N86 A87 E133 |
| 4 | 0.21 | I22 W23 Q24 E33 R34 L35 P36 L48 G49 V50 K51 H52 F65 G66 T67 P79 S80 D678 S679 |
| 5 | 0.21 | L220 P221 S222 Q223 D224 L225 S523 L524 R525 T526 D557 G558 K559 E560 |
| 6 | 0.20 | F588 R640 D642 G646 D647 N648 L649 E651 S654 |
| 7 | 0.19 | K4 W613 S614 L625 V626 D627 F631 Q632 P633 Y669 I670 S671 I688 M689 |
| 8 | 0.08 | G567 G568 Y569 H570 S585 T587 F588 Q590 R619 D620 |
| 9 | 0.07 | D141 A149 V150 R151 S166 I167 S168 E170 W172 K194 |
| 10 | 0.06 | W23 I30 T31 P32 S663 C664 S665 K666 L667 |

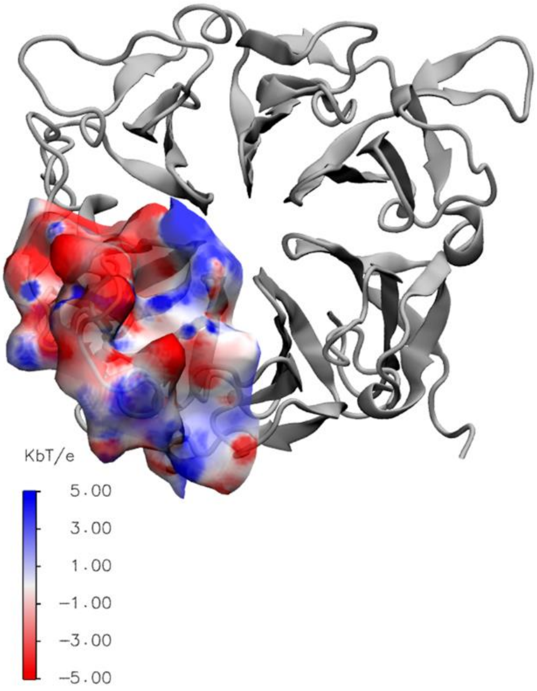


**Fig S6.** Surface electrostatic potential of the putative PIP-binding site computed through APBS software and rendered in VMD.

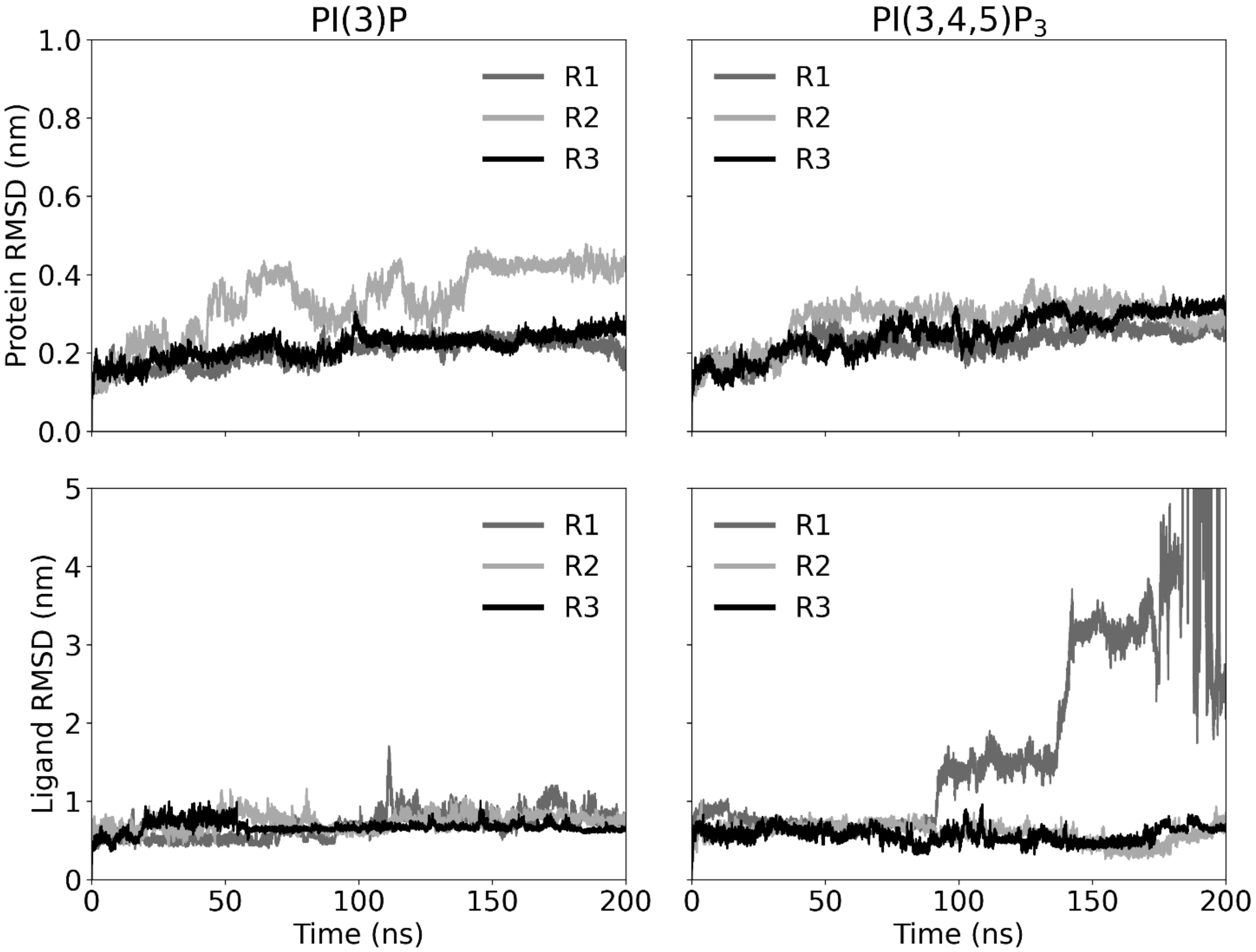


**Fig S7.** Protein and ligand RMSDs for RLD-PI(3)P and PI(3,4,5)$P_3$ systems, where ligand RMSD was obtained fitting the system (protein + ligand) on protein Cα. The three replicas are highlighted in different colors.

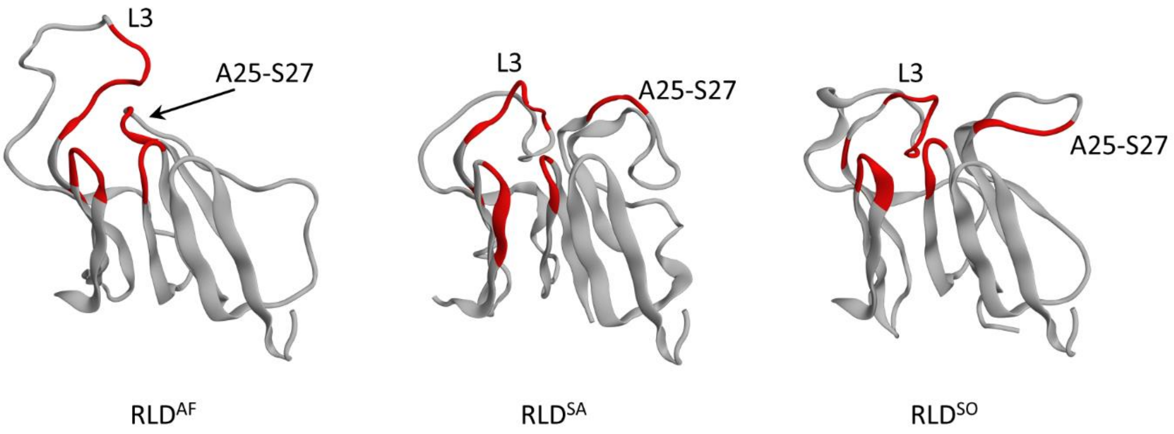


**Fig S8.** Comparison of the putative PIP binding site in $RLD^{SA}$, $RLD^{SO}$, and in the equilibrated $RLD^{AF}$. Residues forming the putative binding pocked are highlighted in red.
